# Do Counter-Narratives Reduce Support for ISIS? Yes, but Not for Their Target Audience

**DOI:** 10.3389/fpsyg.2020.01059

**Published:** 2020-06-11

**Authors:** Jocelyn J. Bélanger, Claudia F. Nisa, Birga M. Schumpe, Tsion Gurmu, Michael J. Williams, Idhamsyah Eka Putra

**Affiliations:** ^1^Department of Psychology, New York University Abu Dhabi, Abu Dhabi, United Arab Emirates; ^2^Department of Psychology, American University in the Emirates, Dubai, United Arab Emirates; ^3^Department of Psychology, Persada Indonesia University, Jakarta, Indonesia

**Keywords:** counter-narratives, violent extremism, need for closure, psychological reactance, ISIS

## Abstract

The purpose of this research is to experimentally test whether counter-narratives are effective to reduce people’s support and willingness to join Islamic State of Iraq and Syria (ISIS). Integrating psychological reactance theory (Brehm, 1966) and need for closure (NFC; Kruglanski, 2004), we predicted that exposing people to counter-narratives when they are at greater risk of radicalization (high NFC individuals) would be counterproductive and enhance their support for ISIS. Participants (*N* = 886 American Muslims) were randomly assigned to a 3 × 3 factorial experimental design varying the source (United States Government, Imam, ISIS defector), and the content (social, political, and religious) of the counter-narrative while comparing these groups to a control message. Results show an overall small positive effect of counter-narratives (β = −0.107, *p* = 0.043), but also evidence for greater support for ISIS in individuals at greater risk of radicalization (β = 0.154, *p* = 0.005). Results also show that the content was more important than the source: A political narrative was the most effective, and this result is consistent across different sources although an ISIS defector is the most effective messenger. These findings challenge the widespread assumption that counter-narratives are effective against violent extremism. In fact, they accelerate the very phenomenon that governments and policy makers are trying to undermine. Therefore, policy makers should avoid including them in their armamentarium to tackle violent extremism.

## Introduction

Despite its recent territorial loss, the Islamic State of Iraq and Syria (ISIS) remains one of the deadliest, most active terrorist groups of our time (Institute for Economics and Peace, 2018). ISIS was catapulted to such notoriety with its effective online propaganda machine capable of flooding the web with slick, extremist digital content (e.g., beheadings, crucifixions, and mass executions), striking fear in the hearts of its enemies and galvanizing new recruits all over the world—up to 30,000 foreign fighters according to the United Nations Security Council (2019). The spread and reach of such communication apparatus have drawn over 60 jihadi movements in 30 countries to pledge allegiance to ISIS (Mohammed, 2014), becoming franchises of a global jihadi brand wreaking havoc in its wake. In the United States, there is a long history of homegrown networks inspired by radical Islam: since 9/11, of the 476 individuals arrested domestically for being connected to jihadist terrorism, the overwhelming majority (75%) has been United States citizens (Williams et al., 2018). Beyond flagging and taking down ISIS’ online content, counterterrorism strategies have relied primarily on reducing the appeal of ISIS’ ideology using counter-narratives, defined as an “intentional and direct communication strategy, within a political, policy, or military context, to discredit messaging of a violent extremist nature” (Ferguson, 2016, p. 8). In a race to win the battle of “the hearts and the minds,” a slew of social media campaigns attempt to critique ISIS’s legitimacy on moral and religious grounds. Some are state-sponsored messages (e.g., United States State Department “Run—do not walk to ISIS land”); others include appeals from religious clerics or ISIS defectors as they are both perceived to be credible voices to challenge ISIS’ narrative.

But how effective are counter-narratives to break the jihadi brand? Despite the widespread assumption among policy makers and practitioners that counter-narratives are successful in preventing violent extremism, scholars have noted the absence of empirical data to substantiate this claim (Ferguson, 2016; Rosand and Winterbotham, 2019)—one of the most glaring gaps in the countering violent extremism literature. Here we provide the first ever experimental test of the effectiveness of counter-narratives to reduce support for ISIS in a sample of American Muslims. To be sure, there is nothing in the Quran that permits terrorism, and the conflation of Islam and terrorism is erroneous. Extremism knows no boundaries; people from any faith (e.g., Christianity, Judaism, etc.), or political affiliation (e.g., left or right wing) can radicalize and support violence to further their ideology. Although non-Muslims could also be influenced by ISIS’ propaganda, the present study surveys American Muslims for the following reasons. First, ISIS’ propaganda directly targets Muslims with the use of Islamic concepts to create a narrative whereby fighting against the West is a religious duty (Ozeren et al., 2018). For example, ISIS encourages Muslims to *hijra* (i.e., migrate for the sake of Allah) to their proclaimed *caliphate* (i.e., an Islamic state under the leadership of a caliph, a person considered the successor to the Prophet Muhammad), and conduct *jihad* (i.e., a struggle or fight against the enemies of Islam). Second, the United States being one of the main targets of Islamist terrorism (Crenshaw, 2010, 2019), ISIS propagandists are keen on recruiting American Muslims because they live in the United States and are, thus, more apt to perpetrate terrorist attacks on American soil (Kruglanski et al., 2019). Third, verses of the Quran are often used in counter-narrative strategies to prevent extremism; knowing whether these verses are actually effective in steering American Muslims away from ISIS would be useful to policy makers and practitioners.

This evaluation is crucial on practical grounds because there has been no systematic effort to provide empirical, let alone experimental, evidence for the effectiveness of counter-narratives. This is a concern given the sizable resources that could potentially be wasted in a strategy with unclear benefits. Furthermore, on theoretical grounds, there are also compelling arguments against the use of counter-narratives. Combining two, hitherto separated, theoretical strands, namely psychological reactance theory and need for closure (NFC), we hypothesized that individuals at greater risk of radicalization (high *need for closure* individuals) who harbor rigid beliefs may display greater psychological reactance when exposed to communication attempts to change their worldview. Consequently, counter-narratives may produce the opposite of the desired effect and increase people’s support for violent extremist groups.

## Psychological Reactance

The present work is grounded in psychological reactance theory (Brehm, 1966), which posits that freedom is a fundamental motivation that generates strong negative reactions when it is threatened or eliminated. The psychological state following a threat to one’s personal freedom is called *reactance*. Psychological reactance has been defined as an aversive state, involving hostile and aggressive feelings as well as negative cognitions (Wicklund, 1974; White and Zimbardo, 1980; Seltzer, 1983; Dillard and Meijnders, 2002; Nabi, 2002; Dillard and Shen, 2005; Quick and Stephenson, 2007). When experiencing reactance, individuals engage in various actions to relieve this feeling and reestablish their freedom. This is why persuasive messages often produce results at odds with their intent (Brehm and Cole, 1966; Burgoon et al., 2002; Schüz et al., 2013). For example, individuals are more likely to engage in behaviors that are forbidden and harbor more positive attitudes toward them than when they are not proscribed (“boomerang effect,” Burgoon et al., 2002; Rosenberg and Siegel, 2018). Likewise, when exposed to counter-attitudinal information, individuals tend to derogate the source of the message or engage in counter-arguing (Hovland et al., 1949; Brehm, 1966; Worchel and Brehm, 1970). Pomeranz et al. (1995) have also shown that people are more likely to dismiss and resist a persuasive appeal if it targets an attitude toward which they are strongly committed.

In the realm of political attitudes, psychological reactance research has shown that, for both conservatives and liberals, exposure to ideologically dissonant information produces political polarization (Nisbet et al., 2015). In a sample of American students, Meirick and Nisbett (2011) found that reactance due to political advertising is “associated directly with more negative cognitive responses, ad, and candidate evaluations and indirectly with lower intention to vote for the candidate supported by the ad” (p. 666). Results from a field experiment also conducted in the United States by Matland and Murray (2013) revealed that social pressure to increase voter turnout backfired and produced anger and hostility toward the message sponsor. Climate change skeptics have also been found to be more likely to display reactance (vs. a control group) when exposed to a message mentioning that there is scientific consensus regarding this topic (Ma et al., 2019). Likewise, research by Zhang (2019) has shown that persuasive appeals in favor of ethical consumption resulted in reactance as evinced by negative appraisal of the source and negative attitudes toward the position advocated.

Extending the foregoing notions to violent extremism, one important question is who may be more likely to display psychological reactance when exposed to a counter-narrative against ISIS? The answer might be individuals with high NFC—people characterized by a desire for firm and unambiguous worldviews (Kruglanski, 2004), who have been shown to be at greater risk of adhering to radical narratives (Webber et al., 2018). Exposing them to information that contradicts their firmly entrenched beliefs may be counterproductive by invigorating their ideological convictions. We now turn to this concept.

## Need for Cognitive Closure

The NFC is defined as a “desire for a firm answer to a question, any firm answer as compared to confusion, and/or ambiguity” (Kruglanski, 2004, p. 6). It’s an epistemic motivation that influences how people process information and make judgments. Individuals with high NFC are driven by obtaining and maintaining closure, meaning that they tend to rapidly “seize” on information permitting a judgment on a given topic and “freeze” on such judgment, thus becoming closed-minded and relatively impervious to new relevant information (Kruglanski and Webster, 1996). Furthermore, NFC is associated with a preference for worldviews that “assume the absolute nature of values and the existence of definite truths” (Golec de Zavala and Van Bergh, 2007, p. 587) because they are stable and predictable belief systems that reduce the probability of having to deal with ambiguity. In contrast, individuals with low NFC eschew binding and definite views.

Empirical research has shown that NFC influences a range of intrapersonal, interpersonal, and group phenomena (see Kruglanski, 2004, for a review) associated with creating consensus and developing a sense of shared reality with other group members (Kruglanski et al., 2006). For example, NFC is associated with exerting and experiencing uniformity pressures (De Grada et al., 1999), agreeing with other group members (Kruglanski et al., 1993), and rejecting group members who express opinions at odds with the group consensus (Kruglanski and Webster, 1991). In the same vein, evidence suggests that NFC is associated with preserving group norms across varying generations of membership (Livi et al., 2007) and preferring unequivocal directives (i.e., harsh power tactics) from leaders (Bélanger et al., 2015a, b).

Radical narratives, such as the one promulgated by ISIS, are attractive to those high in NFC because they depict simplistic, “black-and-white,” Manichean perspectives, whereby good and evil are perpetually locked into an antagonistic struggle, and aggression against the out-group is justified. Furthermore, radical groups are particularly effective in reducing uncertainty given that they are highly structured, have clearly defined goals, and provide a clear sense of purpose and identity (Hogg et al., 2007; Van Den Bos et al., 2007; Dugas et al., 2016). Consistent with this proposition, Webber et al. (2018) found correlational and experimental evidence showing that NFC is related to being unwilling to compromise on important ideological values and endorsing non-normative ideals associated with one’s political party. In large samples of individuals imprisoned for their affiliation with different terrorist organizations (i.e., ISIS, Liberation Tigers of Tamil Eelam), the same authors found that NFC is associated with supporting suicide bombings and armed struggle to further one’s ideology. In sum then, individuals with high NFC are at greater risk of radicalization and potentially more likely to display reactance when exposed to a persuasive message crafted to challenge their firmly entrenched beliefs, destabilizing their sense of certainty and closure. The following study sought to examine this proposition.

## The Present Research

The purpose of this research was to test the effect of counter-narratives to reduce support for ISIS using a 3 × 3 factorial experimental design varying the source of the narrative (United States Government, ISIS defector, and Imam) and the content of the narrative (social, political, or religious narratives) while comparing these groups to a control message. Furthermore, we examine whether the effect of counter-narratives holds for their critical target audience: individuals with high NFC who are at greater risk of radicalization (Webber et al., 2018; Van Den Bos, 2018).

These sources and their content were chosen for theoretical and practical reasons. First, from a theoretical standpoint, research has found that ISIS’ propaganda revolves around “four distinct, yet entangled narratives” (Pellerin, 2016 p. 11): (1) social (establishing a better society), (2) political (bringing a new world order through a global caliphate), (3) religious (using the Quran to legitimize violence), and (4) moral (destroying the West, a symbol of moral decay). We reasoned that, if ISIS has had success recruiting with these themes, they might also be effective in preventing individuals from joining ISIS. In a counter-narrative context, there is always a moral component, an indication that one mode of being is morally reprehensible. For this reason, the social, political, and religious counter-narratives described in the methods section below end with the following moral assertion: “And this is why, violence by the Islamic State (also known as ISIS) is unacceptable.” Second, from a practical standpoint, the counter-narratives in this study model existing counter-narratives used against ISIS (e.g., see Counter-narrative toolkit, n.d.; Extreme Dialogue, n.d.; and Hedayah, n.d.). This was to ensure the content validity of our messages.

## Materials and Methods

### Participants and Procedure

Expecting medium effect sizes and setting power at 0.80, a sample size of 77 participants per experimental condition was suggested using GPower (Faul et al., 2009). Eight hundred eighty-six American Muslims were recruited using Qualtrics^®^ panel service (444 women, 442 men; *M*_age_ = 39.42 years, and SD_age_ = 9.31 years; ethnicity: 68.5% white Caucasians, 1.9% Latinos, 14.1% Black/Africans, 8.7% Asians, 5.4% Arabs, and 1.4% Other; education: 6% high school, 9.1% some college/vocational, 21.6% completed college/vocational, 13.9% some postgraduate studies, and 49.4% completed postgraduate degree; and political preferences: 2.4% far left, 30.4% liberal, 29.9% moderates, 30.3% conservatives, and 7% far right). See Table 1 for demographics.

**TABLE 1:** Demographic profile distribution (*N* = 886).

	**Demographic value**	**Frequency**	**%**
Gender	Male	442	49.9
	Female	444	50.1
Age	18–24	78	8.8
	25–34	127	14.3
	35–49	568	64.1
	50–64	107	12.1
	65+	6	0.7
Ethnicity	Arab	48	5.4
	Asian	77	8.7
	Black	125	14.1
	Caucasian	607	68.5
	Hispanic	17	1.9
	Other	12	1.4
Education	Completed high school	53	6.0
	Some college/vocational school	81	9.1
	Completed college/vocational school	191	21.6
	Some postgraduate	123	13.9
	Completed postgraduate	438	49.4
Political preferences	Far-left	21	2.4
	Liberal	269	30.4
	Moderate	264	29.9
	Conservative	268	30.3
	Far-right	62	7.0

Participants were invited to complete a short survey on political activism. After obtaining their written consent^1^, the importance of religion and participants’ need for cognitive closure was measured, after which they were randomly assigned to one of 10 experimental conditions. Before being exposed to the counter-message, participants were told that “in recent years, the social and political situation in Iraq and Syria has been extremely volatile and unsettling,” that “some people have shared what they think and feel about this conflict,” and that they would read a short paragraph about their opinion and then answer some questions. The dependent variable—support for ISIS—was measured after exposure to the counter-narrative. This online procedure is in line with terrorist organizations that have been using online platforms effectively to recruit, radicalize, and glamorize the use of violence to further their political agendas (Von Behr et al., 2013; Aly et al., 2017). Whether it is through e-magazines, social media, or online forums, virtually all terrorist organizations have moved their communication efforts to cyberspace (Weimann, 2014), and narratives promoting political violence are readily accessible to anyone in a matter of clicks.

### Counter-Narratives

Each counter-narrative was first introduced by the following paragraph:

“In recent years, the social and political situation in Iraq and Syria has been extremely volatile and unsettling. Some people have shared what they think and feel about this conflict. In the following section, you will read a short paragraph about their opinion.”

#### Manipulating the Source

The source of the counter-narrative was specified after the introductory paragraph in the following ways: “an Islamic State defector has said” (in the defector condition), “an Imam has said” (in the Imam condition), and “the United States Government has said” (in the United States Government condition).

#### Manipulating the Content

After mentioning the source of the counter-narrative, the content was manipulated with the following messages inspired by real counter-narrative campaigns.^2^

##### Social counter-narrative

“We have seen it time and again, Islamist groups fighting in Iraq and Syria have committed unspeakable acts of cruelty against innocent people. They destroyed basic infrastructure such as schools, hospitals, sewage treatment plants, power generation, roads, and telecommunication, leaving people scrounging for food and water, and in desperate need of shelter and warm clothing. These ruthless groups have also plundered public resources, prevented old people from going to hospitals, and killed innocent women and children. Innocent civilians have greatly suffered and died because of their reckless actions. And this is why violence by the Islamic State (also known as ISIS) is unacceptable.”

##### Political counter-narrative

“We have seen it time and again, Islamist groups fighting in Iraq and Syria are arrogant opportunists driven by power and only wish to further their own selfish interests. They exaggerate, twist, and turn facts to convince people to fight for them. They can be very charming and persuasive, but they lie, cheat, and fool people into thinking they should obey them and give them money. If that doesn’t work, they’ll take advantage of your weaknesses: loneliness, insecurity, or simple ignorance, to achieve their political and financial goals. And this is why violence by the Islamic State (also known as ISIS) is unacceptable.”

##### Religious counter-narrative

“In the Quran, there is a Surah, “Surah 5, Al-Maida, Ayah 32” that says something important, it says: “*That is why We ordained for the Children of Israel that whoever takes a life—unless as a punishment for murder or mischief in the land—it will be as if they killed all of humanity; and whoever saves a life, it will be as if they saved all of humanity. Although Our messengers already came to them with clear proofs, many of them still transgressed afterwards through the land*.” And this is why violence by the Islamic State (also known as ISIS) is unacceptable.”

#### Control Condition

Participants who were randomly assigned to the control condition were exposed to the following political blurb to ensure that participants across all conditions were exposed to content related to politics, which were also of equal length.

“For thousands of years, the study of political systems has been understood as inseparable from the study of social life as a whole. In the following section, you will read a short paragraph about the study of political systems. After reading it carefully, you will complete a short questionnaire.

A political system is a framework which defines acceptable political methods within a given society. The history of political thought can be traced back to early antiquity, with seminal works such as Plato’s Republic, Aristotle’s Politics and the works of Confucius. A variety of methods are deployed in politics, which include promoting one’s own political views among people, negotiation with other political subjects, making laws, and exercising force, including warfare against adversaries. Politics is exercised on a wide range of social levels, from clans and tribes of traditional societies, through modern local governments, companies and institutions up to sovereign states, to the international level.”

### Measures

#### Need for Cognitive Closure

Participants’ need for cognitive closure (*M* = 5.11, SD = 1.23) was measured using two items taken from Roets and Van Hiel’s (2011) short scale (i.e., “I enjoy having a clear and structured mode of life” and “I dislike unpredictable situations”). The items were correlated (*r*_s_ = 0.41, *p* < 0.001) and were, thus, averaged. Participants were asked to rate the extent to which they agreed to each item on a seven-point scale ranging from one (*Not agree at all*) to seven (*Very strongly agree*).

#### Importance of Religion

The extent to which participants consider religion important (*M* = 5.66, SD = 1.34) was measured with a single item (“Practicing my religious or spiritual beliefs is important for me”) on a seven-point scale ranging from one (*Not agree at all*) to seven (*Very strongly agree*).

#### Support for ISIS

Participants’ support for ISIS (*M* = 3.03, SD = 1.55) was measured using five items (α = 0.91) that were averaged. These items were adapted from previous work on violent extremism (Schumpe et al., 2018a, b). Sample items include “I have a favorable opinion toward the Islamic State (i.e., ISIS),” “I like what the Islamic State (i.e., ISIS) is doing,” and “I would consider joining this group.” Participants were asked to rate the extent to which they agreed to each item on a seven-point scale ranging from one (*Not agree at all*) to seven (*Very strongly agree*).

#### Data Availability

The questions of our survey are included in the Appendix. Furthermore, the data supporting the findings of this study have been deposited in the Open Science Foundation repository: https://osf.io/jsxk6/?view_only=a300e008a90a403c90c14be4d686d211.

## Results

### Counter-Narrative Main Effects

**FIGURE 1:**
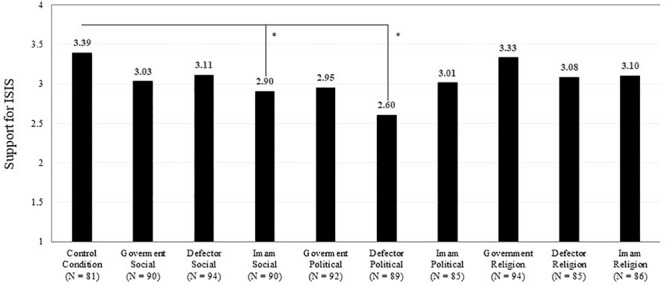
Support for ISIS across experimental conditions (*N* = 886). *p < *0.05.*

We display support for ISIS across all experimental conditions in Figure 1. Table 2 presents four regression models predicting support for ISIS, testing several features of counter-narratives, and adjusting for sociodemographic variables (age, gender, education, political preferences, importance of religion, and ethnicity). The dependent variable, support for ISIS, was not normally distributed; therefore, all analyses were conducted with the bias-corrected and accelerated bootstrap method (5,000 resamples per analysis) to produce accurate estimations of standard errors and 95% confidence intervals (Freedman, 1981; Efron, 1987; Carpenter and Bithell, 2000).

**TABLE 2:** Standardized regression coefficients predicting support for ISIS (*N* = 886).

**Regression models predicting support for ISIS**	**(1)**	**(2)**	**(3)**	**(4)**
Age	0.084	0.075	0.084	0.087
Gender (1 = Male; 2 = Female)	−0.148***	−0.128**	−0.146***	−0.147***
Education	0.220***	0.211***	0.220***	0.221***
Left-right political views	0.296***	0.300***	0.295***	0.296***
Importance of Religion	0.001	–0.020	0.001	–0.001
Ethnicity: White	0.121	0.120	0.121	0.121
Ethnicity: Black	0.133	0.138	0.135	0.134
Ethnicity: Arab	–0.048	–0.052	–0.047	–0.048
Ethnicity: Asian	–0.106	–0.107	–0.105	–0.098
Main effect	−0.107**	−0.112**		
NFC		0.092*		
Main effect*NFC		0.154***		
Source: United States Government			–0.313	
Source: Imam			−0.370*	
Source: ISIS defector			−0.426**	
Content: Social				−0.369*
Content: Political				−0.520***
Content: Religious				–0.223

Adj *R*^2^	0.10	0.11	0.10	0.11
F	11.53	10.70	9.66	10.01

Note: *p < 0.1; **p < *0.05;* and ***p < *0.01*.

Model 1 tests the main overall effect of counter-narratives by comparison to the control group. Taking all sources and narrative contents together, counter-narratives significantly reduce support for ISIS (β = −0.107, *p* = 0.043). A more detailed analysis of variance shows differences between the experimental conditions with only two counter-narratives significantly reducing support for ISIS compared to the control group: a political narrative delivered by an ISIS defector [Tukey HSD mean_diff_ = −0.794, CI (0.33, 1.22)] and a social narrative delivered by an Imam [Tukey HSD mean_diff_ = −0.493 CI (0.03,0.94)]. These two counter-narratives did not significantly differ from one another [Tukey HSD mean_diff_ = −0.30 CI (−0.71,0.11)]. By comparison to the most effective counter-narrative (i.e., political narrative from ISIS defector), a religious counter-narrative delivered by the government was the least effective [Tukey HSD mean_diff_ = 0.730, CI (−1.13, −0.31)].

The overall main effect was moderated by NFC (Model 2). The overall main effect of counter-narratives is strengthened (β = −0.112, *p* = 0.028) when accounting for the interaction with NFC, which produces a reactance effect (β = 0.154, *p* = 0.003; Model 2). There are no differences between the groups for baseline NFC (*F* = 0.981, *p* = 0.454), and the main effect of NFC is marginally positive in increasing support for ISIS (β = 0.092, *p* = 0.057).

Models 3 and 4 focus on the main effects per source and narrative content, respectively. Per source (Model 3), an ISIS defector was the most effective messenger (β = −0.426, *p* = 0.032), followed by an Imam (β = −0.37, *p* = 0.064), with the United States Government being a marginally beneficial source (β = −0.313, *p* = 0.078). Per content (Model 4), political narratives were the most effective (β = −0.520, *p* = 0.009), followed by social narratives (β = −0.369, *p* = 0.059), and no significant effect was produced by religious narratives (β = −0.223, *p* = 0.258).

Regardless of the experimental manipulation, there is, across all models, results indicated that men were more supportive of ISIS than women. Furthermore, political right-wing views and education were positively related to support for ISIS.

### Testing Counter-Narratives for At-Risk Individuals

**FIGURE 2:**
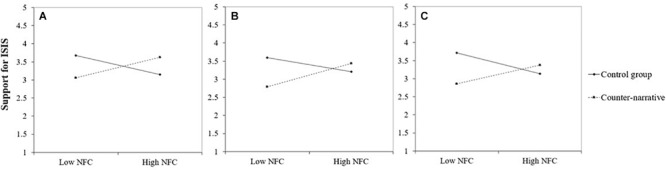
Support for ISIS as a function of Muslims’ need for closure and exposure to a religious counter-narrative (vs. control message) delivered by **(A)** the United States Government, **(B)** an ISIS defector, and **(C)** an Imam. All interactions are significant. Low NFC = −1 SD; High NFC = + 1 SD.

**FIGURE 3:**
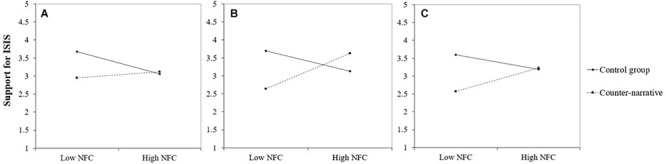
Support for ISIS as a function of Muslims’ need for closure and exposure to a social counter-narrative (vs. control message) delivered by **(A)** the United States Government, **(B)** an ISIS defector, and **(C)** an Imam. All interactions are significant, except **(A)** which, though in the expected direction, was not significant. Low NFC = −1 SD; High NFC = + 1 SD.

Using multiple regression analyses, we then tested whether support for ISIS increases as a function of high (+1 SD) and low (−1 SD) NFC when individuals are exposed to counter-narratives. For each counter-narrative, we entered NFC, the experimental condition (coded 0 = control condition, 1 = counter-narrative), and its interaction term as predictors and controlled for importance of religion. Five of the nine interactions were significant and indicated a boomerang effect; they are described below and presented in Figures 2, 3. The interaction terms that were not significant were the social counter-narrative presented by the United States Government (*p* = 0.10; see Figure 3A) and the political counter-narrative presented by the United States Government (*p* = 0.15), the ISIS defector (*p* = 0.25), and the Imam (*p* = 0.37).

#### Government Religion

Need for closure (β = 0.03, *p* = 0.81) and counter-narrative (β = −0.02, *p* = 0.80) were not associated with support for ISIS, but the interaction was significant (β = 0.27, *p* = 0.02). As displayed in Figure 2A, follow-up simple slope analyses suggest that NFC was positively associated with support for ISIS in the counter-narrative (β = 0.28, *p* = 0.06) but not in the control condition (β = −0.26, *p* = 0.21). The model explained 2% of the variance.

#### Defector Religion

Need for closure (β = 0.07, *p* = 0.60) and counter-narrative (β = −0.13, *p* = 0.27) were not associated with support for ISIS, but the interaction was significant (β = 0.25, *p* = 0.04). As displayed in Figure 2B, follow-up simple slope analyses suggest that NFC was positively associated with support for ISIS in the counter-narrative (β = 0.31, *p* = 0.05), but not in the control condition (β = −0.18, *p* = 0.37). The model explained 2% of the variance.

#### Imam Religion

Need for closure (β = −0.007, *p* = 0.96) and counter-narrative (β = −0.13, *p* = 0.28) were not associated with support for ISIS, but the interaction was significant (β = 0.28, *p* = 0.02). As displayed in Figure 2C, follow-up simple slope analyses suggest that NFC was positively but marginally associated with support for ISIS in the counter-narrative (β = 0.26, *p* = 0.13) but not in the control condition (β = −0.29, *p* = 0.17). The model explained 2% of the variance.

#### Defector Social

Need for closure (β = 0.13, *p* = 0.28) and counter-narrative (β = −0.13, *p* = 0.28) were not associated with support for ISIS, but the interaction was significant (β = 0.39, *p* < 0.01). As displayed in Figure 3B, follow-up simple slope analyses suggest that NFC was positively associated with support for ISIS in the counter-narrative (β = 0.40, *p* = 0.001) but not in the control condition (β = −0.23, *p* = 0.15). The model explained 5% of the variance.

#### Imam Social

Need for closure (β = 0.08, *p* = 0.55) was not related to support for ISIS. However, people exposed to the counter-narrative (*M* = 2.90, SD = 1.54) reported less support for ISIS than people exposed to the control message (*M* = 3.39, SD = 1.60; β = −0.26, and *p* = 0.03). Most importantly, the interaction was significant (β = 0.27, *p* = 0.02). As displayed in Figure 3C, follow-up simple slope analyses suggest that NFC was positively associated with support for ISIS in the counter-narrative (β = 0.27, *p* = 0.04) but not in the control condition (β = −0.17, *p* = 0.29). The model explained 2% of the variance.

## Discussion

Integrating psychological reactance theory and NFC, the purpose of this research was to answer two fundamental questions related to counter-narratives: (1) Can they reduce the appeal of ISIS among American Muslims, and (2) are they effective with at-risk individuals? Although counter-narratives have been part of virtually all counterterrorism strategies around the globe, the present research is the only study testing whether they are effective to mitigate support for a terrorist organization. Overall, our experimental results demonstrate that there is a small but positive effect of counter-narrative on reducing support and willingness to join ISIS. Independently of the content of the counter-narrative, the most effective source was an ISIS defector, followed by an Imam (although marginally); the government as a spokesperson did not produce a significant effect. Independent of the source, the most effective content was the political counter-narrative, followed by the social counter-narrative (although marginally); the religious counter-narrative did not mitigate support for ISIS. Across all experimental conditions, the most successful message was an ISIS defector delivering a political counter-narrative followed by an Imam delivering a social counter-narrative.

Despite these encouraging results, we also found strong support for the notion that counter-narratives often yield the opposite of the intended effect. Indeed, five out of nine counter-messages produced a boomerang effect when shown to their target audience, namely individuals at greater risk of radicalization with high NFC (Hogg et al., 2013; Webber et al., 2018). Our results demonstrate that all counter-narratives with a religious argument backfired regardless of the source of the message. This is an important finding given the widespread assumption that a moderate, mainstream understanding of Islam, especially when articulated by an authoritative religious leader, attenuates the allure of violent extremism (Briggs and Feve, 2013; Braddock and Horgan, 2016). Results did not support that proposition. Likewise, counter-narratives involving a social argument also backfired when delivered by an ISIS defector or an Imam. This shows that highlighting the devastating social effects of ISIS on the Muslim community (i.e., the ummah) does not produce its intended effects despite recommendations from many agencies to use this approach (Jacobson, 2010; National Counter-Terrorism Center, 2011).

The present research, however, is not impervious to methodological limitations. One such limitation consists of the sample that included a large proportion of Caucasian individuals who had completed a postgraduate degree. Future research should attempt to replicate our findings in a different cultural context with a more ethnically diverse sample with a broader educational background. For instance, our findings could be replicated in countries that have produced large numbers of foreign fighters, such as France, Sweden, Belgium, and Norway.

### Policy Implications

Taken together, the present research supports the notion that individuals with high NFC relinquish uncertainty and are, thus, resistant to change and unwilling to compromise on their political convictions (Webber et al., 2018). Attempts to shape their perspective have the unintended effect of strengthening their ideological positions. These findings, thus, challenge the widely held assumption that the appeal of violent extremism among vulnerable individuals will decrease if they are exposed to narratives intended to break the jihadi brand. Consequently, the fundamental practical implication of this work is that practitioners, NGOs, and governments should refrain from using counter-narrative strategies to counter violent extremism.

Furthermore, the fact that the social narrative delivered by an Imam generally produced positive results but backfired when shown to high-NFC individuals, suggests that policy makers and practitioners should choose their target audience carefully and disseminate their counter-narratives through narrowcasting (as opposed to broadcasting) or perhaps even one-to-one conversation to avoid exposing segments of the population susceptible or sympathetic to narratives of violent extremism. Interestingly, however, no backfire effect was observed for political counter-narratives, and this begs future research to examine why some counter-narratives provoke more reactance than others among higher risk individuals. One possible explanation is that revealing that ISIS exploits people for its own political agenda creates a state of disillusionment with the terrorist group and is, thus, more effective to neutralize the jihadi narrative. Future research should clarify these issues and examine the effect of counter-narratives across cultures to increase the generalizability of the present findings.

### Theoretical Implications

The present research, borne out of the integration of psychological reactance theory and NFC, affords new insights for each line of work. One of the main contributions of this research is to show that psychological reactance is relevant to the study of terrorism—a critical point given that one of the primary objectives of this field of inquiry is to craft effective methods to convince individuals to abandon violent means in the pursuit of their political or religious goals (see Bélanger, 2017). This contribution is significant considering that scholars have observed that using counter-narratives to prevent violent extremism “is built on very shaky theoretical and empirical foundations” (Glazzard, 2017, p. 1). And, indeed, the present research demonstrates that counter-narratives can be counterproductive by creating reactance and increasing the appeal of violent extremism among individuals who are at greater risk of radicalization. Although these results challenge the widespread assumption that counter-narratives are effective against violent extremism, they provide support for the tenets of psychological reactance theory and mirror the findings in public health research whereby health promotion messages are often shown to increase the behavior they are intended to mitigate (e.g., Ruiter et al., 2001; Grandpre et al., 2003; Schüz et al., 2013).

The second theoretical contribution of this research is to demonstrate that the NFC is related to psychological reactance—a relationship that hasn’t been documented in prior work. Indeed, by showing that the NFC creates a boomerang effect, we provide evidence that individuals with entrenched beliefs are more likely to resist persuasive appeals to a higher degree. This contribution is meaningful because psychological reactance theory predicts that the magnitude of reactance increases as a function of the importance of the freedom that is threatened, which is typically manipulated by increasing the importance of a behavior or an attitude or, in Brehm and Brehm’s (1981) terminology, “varying [the] magnitude of need” (1981, p. 41). Here, we show that one such need is NFC, and it reflects the degree to which people want to preserve their belief systems to avoid uncertainty. The greater such need, the greater the reactance to persuasive appeals. All in all, the present work contributes to psychological reactance theory by showing that NFC produces a counterforce motivating people to reassert their belief in an ideological system that affords them to maintain closure across time and contexts.

## Conclusion

There is a consensus that eradicating violent extremism requires a long-term investment in structural, complex, multilayered interventions from education to social and economic development (Global Counterterrorism Forum, 2012). Concomitantly, there is also the need to act in the short term with strategies that can assist in preventing or countering violent extremism in more immediate timespans. The threat from terrorist propaganda is real, and counter-narratives are the cornerstone short-term intervention in the fight against violent extremism. However, the urgency to deploy communication strategies to attenuate the appeal of ISIS should not be an excuse to avoid rigorous standards to produce evidence-based policies because the risk of backfiring and accelerating further radicalization is also real and threatens public safety. Until future research can further assess these effects, we suggest counter-narratives should be used by practitioners and policy makers’ campaigns only after careful consideration.

## Data Availability Statement

The datasets generated for this study are available on request to the corresponding author.

## Ethics Statement

The studies involving human participants were reviewed and approved by New York University Abu Dhabi, Protocol titled Dehumanization and Violence (no. 043-2017). The patients/participants provided their written informed consent to participate in this study.

## Author Contributions

JB was involved in all steps of this work from conceptualization to final manuscript submission. CN was involved in data analysis and manuscript editing. BS was involved in manuscript editing. TG, MW, and IP was involved in manuscript editing.

## Conflict of Interest

The authors declare that the research was conducted in the absence of any commercial or financial relationships that could be construed as a potential conflict of interest.

## Footnotes

1 The research protocol was approved by New York University Abu Dhabi Institutional Review board (protocol #043-2017).

2 The social, political, and religious counter-narratives were inspired by the following sources, respectively: https://www.military.com/video/operations-and-strategy/terrorism/welcome-to-the-islamic-state-land/3775821940001 https://www.youtube.com/watch?v=3Lb7J6WDZpw https://www.youtube.com/watch?v=ceaaS8-mj-k

## Appendix

Each measure was answered on the following seven-point scale:

1. Not agree at all
2. Very slightly agree
3. Slightly agree
4. Moderately agree
5. Mostly agree
6. Strongly agree
7. Very strongly agree

### Need for Closure

Read each of the following statements and decide how much you agree with each according to your beliefs and experiences.

1. I enjoy having a clear and structured mode of life
2. I dislike unpredictable situations.

### Importance of Religion

While thinking of Islam, please indicate your level of agreement with the item below.

1. Practicing my religious or spiritual beliefs is important for me.

### Support for ISIS

The following questions pertain to the Islamic state (also known as ISIS). Read each of the following statements and decide how much you agree with each according to your beliefs and experiences.

1. I like the Islamic State (i.e., ISIS) very much.
2. I have a favorable opinion toward the Islamic State (i.e., ISIS).
3. I like what the Islamic State (i.e., ISIS) is doing.
4. I would consider joining this group.
5. I think what the Islamic State (i.e., ISIS) is doing is morally reprehensible.
